# Vaping and Tuberculosis: An Overlooked Risk in High‐Burden South Asian Countries

**DOI:** 10.1002/puh2.70191

**Published:** 2026-02-09

**Authors:** Hafsa Ali, Rimsha Riaz, Maheen Kashif, Syeda Nurjis Fatima, Ahmed Ali

**Affiliations:** ^1^ Sindh Medical College Jinnah Sindh Medical University Karachi Pakistan; ^2^ Karachi Medical & Dental College Karachi Pakistan; ^3^ Faculty of Medicine University of Khartoum Khartoum Sudan

## Abstract

Tuberculosis (TB) is an infectious disease that remains one of the most pressing threats to public health, especially in South Asian countries. Despite being curable, it is a major cause of morbidity and mortality, driven by factors such as poverty, lack of nutrition, HIV infection, and overcrowding of individuals. Among modifiable risk factors, tobacco smoking has long been established as a major contributor to TB incidence. However, the increasing use of vapes/e‐cigarettes, perceived as a safer alternative to traditional smoking, can pose as an underrecognized threat to TB control, particularly in high‐burden regions. Vaping exposes individuals to nicotine, flavoring agents, and toxic substances that compromise lung immunity. Evidence shows that vaping impairs alveolar macrophage function, reduces cytokine signaling, and suppresses defense peptides in the host. All of these factors enhance *Mycobacterium tuberculosis* pathogenesis. Radiologically, vaping‐induced injury can resemble features of military TB, such as ground‐glass opacities, complicating diagnosis and eventually leading to misdiagnosis. Despite these concerns, South Asian countries fail to enforce strategies to ban the use of e‐cigarettes. To address this important public health concern, vaping must be incorporated into TB risk assessments and recognized as a modifiable risk factor to strengthen public health efforts and accelerate progress towards TB elimination in South Asian countries.

## 1 Introduction

Pulmonary tuberculosis (TB) is a rising infectious disease caused by the organism *Mycobacterium tuberculosis*. It is an important public health concern, particularly in South Asian countries like Pakistan, India, and Bangladesh. Despite being curable and preventable, the disease claims to be a major healthcare and socio‐economic challenge. According to the World Health Organization (WHO), it is estimated that 56% of the global TB burden in 2024 lay on five countries: India, Indonesia, China, the Philippines, and Pakistan, of which two major contributors were South Asian countries, India (26%) and Pakistan (6.3%) [1]. In 2022, the WHO estimates reported that more than 4.8 million people contracted the infection, out of which more than 600,000 died in the region, which accounts for more than 50% of the global TB‐related deaths [2]. Factors that drive TB prevalence in these countries include poverty, malnutrition, overcrowding, HIV infection, and the absence of a robust disease surveillance system. One very important risk factor for developing TB is smoking, responsible for more than 20% of the TB cases worldwide [1, 3]. The main component accountable for immune dysfunction is nicotine [3].

Nowadays, the trend of using e‐cigarettes/vapes as a modern smoking alternative has escalated, especially among young people. The aim is to provide a less irritating substitute to combustible cigarettes for the people who want to quit smoking. The innovative flavors and packaging, social trends, and lack of regulation all contribute to its popularity, leading to a perception that vaping is safer than smoking. Studies, on the other hand, show that nicotine exposure is nearly similar to conventional smoking [4]. A survey comprising 121 countries was conducted in 2015, which concluded that 90% of smokeless tobacco users live in South Asia [5]. E‐cigarettes contain toxins like propylene glycol, nicotine, and flavoring agents. These can cause inflammation and high oxidative stress, damaging DNA, leading to lung injuries. The lungs become weak to fight infections, increasing susceptibility to TB and contributing to the severity of active disease [4]. A detailed mechanism is explained in the next section. Therefore, this article aims to shed light on an emerging public health issue that vaping could be a hidden risk factor for the development of TB, especially in high‐burden countries, where vaping is increasingly popular.

## 2 Linking Mechanism

Vaporizers and e‐cigarettes that are believed to be a good replacement for traditional cigarettes are, in fact, an underrecognized source of compromised lung health. One of the most preventable yet alarming infections, *M. tuberculosis* (TB), has recently evolved to be linked with it. The main damaging culprits are the loaded nicotine, flavoring agents, and other toxins, having a direct impact on the THP‐1 (alveolar‐macrophage system), reducing phagocytosis [6]. This remarkably increases the virulence of *Mycobacterium* that disrupts the receptor mechanisms of the lung, for instance, TLR2, TLR4, and NOD2, which are solely required for detecting and signaling pathogenic activity [7]. Additionally, due to nicotine uptake, the TLR4 and NOX2 axis is abruptly deviated, downgrading the oxidative bactericidal properties of reactive oxygen species (ROS) [7]. Furthermore, e‐cigarettes have a suppressing influence on peptides like LL‐37 and gamma defensins, as well as surfactant proteins (A), crucial for controlling virulence via phagocytosis. Cytokines produced that are damaged, along with TNF‐alpha and IL‐6, add to the weakening of the immunocompetency of the lungs [6]. Lastly, when *Mycobacterium* gets completely exposed to the lung surface, it results in widespread destruction, leading to diffused alveolar hemorrhage to lipoid pneumonia. Recent diagnostic research reveals CT scans with bilateral micronodular opacities, subpleural sparing, and septal thickening, the characteristic findings of e‐cigarette or vaping product use‐associated lung injury (EVALI) that worsen the mucosal barriers, creating a gateway for *Mycobacterium* and the development of latent TB that can further activate miliary TB [8]. This contagious activation of military TB can be a threat to vulnerable TB‐epidemic populations.

## 3 Diagnostic Overlap and Clinical Confusion

In TB‐endemic regions, the occurrence of EVALI possesses several diagnostic challenges; it commonly presents with symptoms of respiratory concerns like dyspnea, hoarse cough, chest pain, and possibly gastrointestinal symptoms as well, such as nausea and vomiting, which all closely relate to miliary TB. Radiologically, EVALI appears to have a ground‐glass opacity feature, which occasionally could resemble miliary nodular patterns [9]. Moreover, with less than 50% of practitioners adhering to sputum microscopy procedures, diagnosis is often made on radiological signs (x‐ray) due to financial constraints, increasing the risk of misdiagnosis and contributing to a diagnostic overlap [8].

The clinical consequences of such incorrect diagnoses are severe. Patients with EVALI misdiagnosed as TB may endure months of unnecessary anti‐TB medication, which can lead to severe hepatotoxicity, gastric distress, neuropathy, and skin rashes. Furthermore, nonessential use of such medications can potentially cause drug resistance in case *M. tuberculosis* actually infects the person later on in life [10]. Similarly, a person with TB, specifically military TB, misdiagnosed as EVALI, would not receive conventional drug therapy for the treatment of the infection, leading to mortality and spread of the disease [11].

## 4 Epidemiological and Real‐World Evidence Linking Vaping and TB

Population‐level studies directly quantifying the association between TB and vaping remain limited, mainly due to the relatively recent rise in the incidence of vaping. However, robust evidence consistently demonstrates the strong relationship between tobacco smoking and TB infection, offering a reliable comparator given the shared exposure to nicotine and overlapping immunomodulatory effects between vaping and TB. Many meta‐analyses have proven that smoking increases the likelihood of latent TB infection, establishing a dose–response relationship between TB and active smoking. Furthermore, progression to active disease and disease severity leading to mortality are common findings [12]. In a recent meta‐analysis and systematic review of 26 studies, Lin et al. further support the findings, strongly establishing tobacco smoking as a risk factor for active disease by demonstrating twice as much TB occurrence in smokers compared to nonsmokers [12, 13]. Vidyasagaran et al. conducted a meta‐analysis, including 28 studies that quantified the chances of TB reoccurrence and relapse in people with current intake of tobacco (RR, 1.95; 95% CI, 1.59–2.40; *I* ^2^ = 72%) and in participants who used tobacco previously (RR, 1.84; 95% CI, 1.21–2.80; *I* ^2^ = 74%). Increased mortality in current users of tobacco was also a significant finding of the study (RR, 1.51; 95% CI, 1.09–2.10; *I* ^2^ = 87%) [14]. This well‐established statistical relation between TB and smoking tobacco, combined with shared exposure to nicotine and effects on the immune system, supports the consideration of vaping as a plausible emerging risk factor for TB.

## 5 Policy Implications and Gaps

The escalating impact of flavored e‐liquids seems particularly appealing to adolescents, and they are now part of a trend due to their propaganda of sponsorship and compulsive advertisements. As reported by a London hospital study stating that 65% of their TB patients are South Asian‐born. It is also the region with the highest production and usage of tobacco products. The liability for addressing these circumstances falls on the government, which can combat the issue by implementing advocacy campaigns and reducing access to vaping products [15, 16].

Pakistan's Tobacco Control Strategy does not encompass vaping in its anti‐smoking campaigns and solely accentuates tobacco products in its underlying laws by unrestricted publicity, advertisement, and sale services, highlighting an annual growth rate of 1.39% and an estimated profit of $77.2 million in 2024 from e‐cigarettes. Conversely speaking, India has seen more than a 50% rise in the sales of vapes from 1.6 to 3.3 million ever since it officially banned the use of e‐cigarettes and vapes in 2019. Bangladesh planned to restrict use, but no official statistics are available [16].

These existing schemes notably focus on smoking cessation therapies, primarily tobacco. These clinical observations emphasize the correlation between vaping and smoking, especially in those containing tetrahydrocannabinol. Although efforts are being made to incorporate these less risky products into tobacco control programs, as of now, there are no comprehensible results, thus reflecting poor guidelines and the detrimental effects of tobacco [16].

## 6 Recommendation and Call to Action

To evaluate the link between TB and the use of e‐cigarettes, some measures should be taken. Consideration of vaping should be an essential element in the clinical history of a TB patient to evaluate the degree of damage. Vaping impairs phagocytic activity and suppresses immune response; thus, health education awareness campaigns should include its immunosuppressive effect, especially in high‐risk TB communities [6]. To access the outcome and range of severity of TB between vapers and non‐vapers government should contribute via funding region‐specific long‐term research. The lack of research on this important association poses a barrier, and the national TB control program should focus more on collecting data to clarify the role of e‐cigarettes in the progression and transference of disease.

Moreover, the use of e‐cigarettes should be limited; the governments should enforce strict regulations on the sale and use of vaping products to prevent an e‐cigarette epidemic. Accessibility should be highly discouraged in the younger population, and tariffs should be placed on their use. The WHO recommends a tariff of 75% on the sale price to make a strong strategy [15, 16].

## 7 Conclusion

E‐cigarettes and vaporizers linked to serious respiratory conditions like TB have become an emerging yet underestimated epidemic, especially in high‐burden regions like South Asia that lack vaping‐related awareness. Vaping has adverse effects on the immunocompetency of lungs due to impairment of the functionality of lungs, including their macrophage system and alveolar structure, which in turn increases the virulence of *M. tuberculosis*. Furthermore, similarities in the diagnostic features of EVALI and other respiratory disorders result in misdiagnosis and late detection of EVALI. To reduce this, awareness of vaping hazards should be provided to the general population, especially by medical physicians who suspect a smoking history in patients. Recognizing e‐cigarettes as a risk factor for TB, current public health strategies can be significantly improved and result in a reduction of this preventable illness in susceptible individuals.

## Author Contributions

**Hafsa Ali:** project administration; supervision; validation; writing – original draft; writing – review and editing. **Rimsha Riaz:** writing – original draft. **Maheen Kashif:** writing – original draft. **Syeda Nurjis Fatima:** writing – original draft. **Ahmed Ali:** writing – review and editing.

## Funding

The authors have nothing to report.

## Ethics Statement

Ethical approval was not required for this study.

## Conflicts of Interest

The authors declare no conflicts of interest.

## Data Availability

Data sharing does not apply to this article as no new data were created or analyzed in this study.
